# ApoE4 Induces Synaptic and ERG Impairments in the Retina of Young Targeted Replacement ApoE4 Mice

**DOI:** 10.1371/journal.pone.0064949

**Published:** 2013-05-31

**Authors:** Ran Antes, Raaya Ezra-Elia, Dov Weinberger, Arie Solomon, Ron Ofri, Daniel M. Michaelson

**Affiliations:** 1 Department of Neurobiology, Tel Aviv University, Tel Aviv, Isreal; 2 Koret School of Veterinary Medicine, Hebrew University of Jerusalem, Rehovot, Israel; 3 Department of Ophthalmology, Rabin Medical Center, Petach Tikva, Israel; 4 Goldschleger Eye Research Institute, Tel Aviv University, Tel Hashomer, Israel; Institut de la Vision, Paris, France

## Abstract

The vertebrate retina, which is part of the central nervous system, is a window into the brain. The present study investigated the extent to which the retina can be used as a model for studying the pathological effects of apolipoprotein E4 (apoE4), the most prevalent genetic risk factor for Alzheimer's disease (AD). Immunohistochemical studies of retinas from young (4 months old) apoE4-targeted replacement mice and from corresponding mice which express the AD benign apoE3 allele, revealed that the density of the perikarya of the different classes of retinal neurons was not affected by apoE4. In contrast, the synaptic density of the retinal synaptic layers, which was assessed immunohistochemically and by immunoblot experiments, was significantly lower in the apoE4 than in the apoE3 mice. This was associated with reduced levels of the presynaptic vesicular glutamatergic transporter, VGluT1, but not of either the GABAergic vesicular transporter, VGaT, or the cholinergic vesicular transporter, VAChT, suggesting that the glutamatergic nerve terminals are preferentially affected by apoE4. In contrast, the post synaptic scaffold proteins PSD-95 and Gephyrin, which reside in excitatory and inhibitory synapses, respectively, were both elevated, and their ratio was not affected by apoE4. Electroretinogram (ERG) recordings revealed significant attenuation of mixed rod-cone responses in dark-adapted eyes of apoE4 mice. These findings suggest that the reduced ERG response in the apoE4 mice may be related to the observed decrease in the retinal nerve terminals and that the retina could be used as a novel model for non-invasive monitoring of the effects of apoE4 on the CNS.

## Introduction

Alzheimer’s Disease (AD), the most prevalent form of dementia in the elderly, is characterized by cognitive decline and by the occurrence of brain senile plaques and neurofibrillary tangles (NFT), as well as by synaptic and neuronal loss [1]–[3]. Synaptic dysfunction and loss is the earliest histological neuronal pathology in AD [4]–[7] and is also apparent in mild cognitive impaired (MCI) individuals prior to their conversion to clinical AD [8]. Furthermore, synaptic degeneration evolves in a distinct spatio-temporal pattern [9] which, like NFT, radiates from the entorhinal cortex to the hippocampus and subsequently to the rest of the brain [10]. Although AD is not a single neurotransmitter disease, it is associated with distinct and specific neuronal and synaptic impairments. Accordingly, the cholinergic and glutamatergic systems are particularly susceptible to AD [11], [12], whereas the GABAergic system is more resilient and relatively spared [13], [14]. The mechanisms underlying synaptic degeneration in AD and its neuronal specificity are not fully understood.

Genetic and epidemiological studies revealed allelic segregation of the apolipoprotein E (apoE) gene to families with a higher risk of late onset AD and of sporadic AD [15]–[17]. There are three major alleles of apoE, termed E2 (apoE2), E3 (apoE3), and E4 (apoE4), of which apoE4 is the AD risk factor. The frequency of apoE4 in sporadic AD is >50%, and it increases the risk for AD by lowering the age of onset of the disease by 7 to 9 years per allele copy [16]. Histological and biochemical studies of AD brains and brains of transgenic mice that express human apoE3, the AD benign apoE allele, and apoE4, revealed that apoE4 is associated with decreased neuronal plasticity [18] and with synaptic pathology [19]–[24]. The mechanisms underlying the effects of apoE4 in the brain and their neuronal and synaptic specificity are not known. Progress in this regard is hampered by the complexity of the brain and the multitude of its neuronal populations.

The vertebrate retina, which originates as an outgrowth of the developing brain, is part of the central nervous system and can be considered a specific part of the brain. The retina is a layered structure with several layers of interconnected neurons. These include the outer nuclear layer (ONL), which contains the cell nuclei of the photoreceptor cells. These cells connect via the bipolar cells that reside in the inner nuclear layer (INL), to the ganglion cell layer (GCL) whose axons project from the retina via the optic nerve to the brain. The synaptic connections between these neurons form two layers. Accordingly, the outer plexiform layer (OPL) contains the synapses linking the ONL to the INL, whereas the inner plexiform layer (IPL) contains the synaptic connections between the INL and GCL. Laterally connecting horizontal cells that integrate and regulate the input from the photoreceptors are located in the OPL, while the amacrine cells that modulate the output of the bipolar cells to the GCL are found in the IPL. This neuronal architecture renders the retina most suitable for studying the susceptibility of distinct CNS neuronal classes to insults.

A growing body of evidence suggests that AD is associated with visual dysfunction and retinal pathology. These impairments include loss of ganglion cells [25], [26], as well as the accumulation of Aβ-containing deposits termed drusen [27]. The effects of apoE4 on the retina have also been studied. The literature in this regard is, however, sparse and focuses on diseases other than AD. Accordingly, it has been suggested that apoE4 is a risk factor for macular edema in type 2 diabetes [28] and that, surprisingly, it is protective of age-related macular degeneration (AMD) [29], [30]. Animal model studies utilizing aged apoE4-targeted replacement mice, which were maintained on a high-fat cholesterol-enriched diet, revealed pathological changes that mimic those associated with human AMD. These observations provide a proof of principle that retinal neurons, like brain neurons, are differentially affected by the different human apoE genotypes. Additional studies are needed for unraveling how different apoE isoforms affect the retina under normal and diseased conditions and for elucidating the mechanisms that underlie them.

We presently employed the retina as a model for studying the neuronal and synaptic specificity of the pathological effects of apoE4 in young targeted replacement mice and showed that they correlate with the corresponding effects of apoE4 in the brain.

## Materials and Methods

### Ethics Statement

The experiments were approved by the Tel-Aviv University Animal Care Committee (Permit # L-11-041). Every effort was made to reduce animal stress and to minimize animal usage.

### Transgenic Mice

ApoE-targeted replacement mice, in which the endogenous mouse apoE was replaced by either human apoE3 or apoE4, were created by gene targeting, as described in [31]. The mice used were purchased from Taconic (Germantown, NY). Mice were back-crossed to C57BL/6J (Harlan 2BL/610) for ten generations and were homozygous for the apoE3 (3/3) or apoE4 (4/4) alleles; hereafter, these mice are referred to as apoE3 and apoE4 mice, respectively. The apoE genotype of the mice was confirmed by PCR analysis, as described previously [32], [33]. All the experiments were performed on age-matched male animals (4 months of age).

### Preparation of Frozen Sections for Histology

Mice were euthanized by cervical dislocation and their eyes were enucleated. The eyes were fixed in 4% paraformaldehyde (PFA) in PBS for 1 hr, after which the cornea was dissected and the lens was removed. The eye cups were then fixed in 4% PFA in PBS for an additional hour, washed in PBS, and then placed in 15% sucrose for 1 hr followed by 30% sucrose overnight. The fixed eyes were then embedded in Tissue-Tek OCT (Optimal Cutting Temperature) compound (Sakura Finetek, Torrance, CA, USA) for 1 hr and frozen on dry ice. The eye cups were serially dissected into 16 µm sagittal sections, using a cryostat at −20°C, and then mounted on slides. The mounted sections were then used for histological examination as outlined below.

### Hematoxylin and Eosin Staining

The slides were first incubated for 8 min in Hematoxylin (Sigma), washed with water and then with 1% HCl in 70% ETOH to remove excess dye. They were then incubated for 7 min in 1% Eosin (Sigma), washed in running tap water, and mounted with histomount (Invitrogen). The sections were viewed using a Zeiss light microscope (Axioskop, Oberkochen, Germany) interfaced with a CCD video camera (Kodak Megaplus, Rochester, NY, USA). Images of stained retinas were obtained at X40 magnification. The width of each retinal layer was quantified and analyzed using the Image-Pro Plus System for image analysis (v. 5.1, Media Cybernetics). 8–10 retinas of apoE3 and apoE4 retinas (three sections of each retina per slide) were stained and analyzed together. Each such staining was performed on retinas from two different sets of mice.

### Immunofluorescence and Confocal Microscopy

Retinal slices were washed X3 in PBS, after which they were blocked using PBS with 0.2% Tween and 0.2% Gelatine (PBS-TG) for 2 hrs and washed with PBS. The slides were then incubated with the indicated primary antibody overnight at 4°C, after which they were washed (X3 with PBS-TG followed by X3 with PBS), incubated with secondary antibody for 2 hr at room temperature, and washed again (X3 with PBS-TG followed by X3 with PBS). The immunostained sections were then covered with coverslips utilizing Fluoroshield Mounting Medium that contained the nuclear stain DAPI (Abcam).

The sections were immunostained with the following primary antibodies:

Photoreceptors - rabbit anti-recoverin 1∶1000 (Chemicon); Amacrine cells - rabbit anti-Pax6 1:400 (Covance), Bipolar cells - sheep anti-CHX10 1:1000 (Xalpha), Rod bipolar cells - rabbit anti-PKCα 1∶1200 (Santa cruz); Horizontal cells - rabbit anti-Calbindin 1∶1000 (Chemicon), Synapses - mouse anti-Synaptophysin 1∶250 (Sigma); Guinea pig anti-VGluT1 1:2000 (Millipore) mouse anti-VGaT 1∶250 (Synaptic systems) and rabbit anti-VAChT 1∶200 (Synaptic systems), which are markers for glutamatergic, GABAergic and cholinergic nerve terminals, respectively, Goat anti-human apoE 1∶5000 (Calbiochem) and mouse anti-Glutamine Synthetase (GS) 1∶300 (Millipore) which is a marker for Muller cells.

The sections were visualized using a Confocal scanning laser microscope (Zeiss, LSM 510). Images (1024×1024 pixels at X25 or X40 magnification) were obtained by averaging 4 scans per slice. The intensities of immunofluorescence staining, expressed as the percentage of the area stained above a fixed threshold background, were calculated utilizing the Image-Pro Plus System (version 5.1, Media Cybernetics) as previously described [34]. 8–10 retinas of apoE3 and apoE4 retinas (three sections of each retina per slide) were stained and analyzed together. Each such staining was performed on retinas from two different sets of mice. All the images for each immunostaining were obtained under identical conditions, and their quantitative analyses were performed with no further handling.

### Western Blot (WB) Analysis

Mice were euthanized by cervical dislocation and their retinas were rapidly excised and frozen in liquid nitrogen. The retinas were then homogenized in 200 µl 10 mM Tris HCl pH 7.6, which contained NaCl 0.15 M, Triton 1%, Deoxicholic acid 0.5%, SDS 0.1% PMSF 0.3 mM, DTT 0.1 mM, Sodium Orto Vanadat 0.2 mM as well as Protease Inhibitor Cocktail (Calbiochem). The homogenates were then aliquoted and stored at –70°C. The samples were boiled for 10 min prior to gel electrophoresis, after which the electrophoresis and immunoblot assays were performed utilizing the following antibodies: Rabbit anti-Synaptophysin 1∶5000 (Santa Cruz), mouse anti-VGluT1 1:100 (Millipore), mouse anti-VGaT 1∶1000 (Millipore), goat anti- apoE 1∶10000 (Millipore), rabbit anti-PSD-95 1:500 (abcam), rabbit anti-Gephyrin 1∶1000 (abcam) and mouse anti-GAPDH 1∶1000 (abcam). Protein concentration was determined utilizing the BCA protein assay kit (Pierce).

The immunoblot bands were visualized utilizing the ECL chemiluminescent substrate (Pierce), after which their intensity was quantified using EZQuantGel software (EZQuant, Tel Aviv, Israel). GAPDH levels were employed as gel loading controls and the results are presented relative to the apoE3 mice.

### Electroretinography (ERG)

Recordings were conducted in a shielded room isolated from light and electrical noise. Animals were dark adapted overnight and their pupils were dilated with tropicamide 0.5% 15 minutes before recording. Animals were anesthetized with an intraperitoneal injection of ketamine (80 mg/kg) and xylazine (16 mg/kg). To maintain a normal body temperature at 37°C, a heating table was used during anesthesia. To improve conduction, the recorded eyes were kept moist with a drop of hydroxymethylcellulose (1.4%). Signals were recorded using a gold loop wire. Subcutaneous needles served as reference and ground electrodes, and were placed at the middle of the forehead and in the base of the tail, respectively. Both eyes were recorded at a random order Impedance was kept under 7 KΩ. All recordings were done using Handheld Multi-species Electroretinography system (HMsERG, Ocuscience, Missouri, USA), with a bandpass of 0.3–300 Hz. Intensity-response curves were recorded using 13 steps of increasing flash intensity (0.00003, 0.0001, 0.0003, 0.001, 0.003, 0.01, 0.03, 0.1, 0.3, 1, 3, 10, and 25 cd*s/m^2^). At the first three intensity levels (0.00003, 0.0001 and 0.0003 cd*s/m^2^) ten flashes, presented at 0.5 Hz, were averaged. At the next three intensity levels (0.001, 0.003, and 0.01 cd*s/m^2^), four flashes, presented at 0.2 Hz, were averaged. At the next five intensity levels (0.03, 0.1, 0.3, 1, and 3 cd*s/m^2^), four flashes, presented at 0.1 Hz, were averaged and at the last two intensity levels (10 and 25 cd*s/m^2^), one flash was presented. Next, photopic flash ERG responses were recorded after 10 minutes of light adaptation (30 cd/m^2^) using the HMsERG background light. Responses to standard and high-intensity flashes (3 and 10 cd*s/m^2^, an average of 32 flashes at 2 Hz) were recorded. Finally, cone flicker responses to the standard and high-intensity flashes were recorded (3 and 10 cd*s/m^2^, an average of 128 flashes at 31.25 Hz).

A- and b-wave amplitudes were measured from baseline to the first trough and from that trough to the next positive peak, respectively. The kinetics of the responses was measured in terms of implicit times (IT), which is the corresponding time interval between the stimulus onset to the trough or to the positive peak.

### Statistical Analysis

Values are presented as mean ± SEM. Student’s *t-*tests were performed to compare mean values of the apoE3 and apoE4 mice. Significant difference between groups was set at *P*<0.05.

## Results

**Figure 1:**
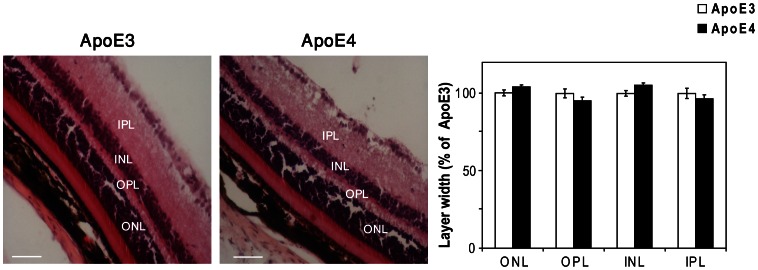
General morphology of the retinas of young apoE4 and apoE3 mice. Representative retinal sections of apoE3 and apoE4 mice stained with hematoxylin and eosin are depicted on the left. Quantification of the thickness of the outer and inner nuclear layers (ONL and INL), which respectively, contain photoreceptors and bipolar cells, and of the outer and inner synaptic plexiform layers (OPL and IPL) of the apoE3 and apoE4 mice is shown on the right. The results (mean ± SEM, n = 10) of the apoE4 mice are presented relative to the apoE3 mice whose values were set as 100%.

Histological examination revealed that the general retinal morphology and thickness were similar in the apoE3 and apoE4 mice. Furthermore, the thickness of the outer and inner nuclear layers in which the photoreceptor and bipolar cells reside and of the synaptic outer and inner plexiform layers were also unaffected by the apoE genotype (Figure 1).

**Figure 2:**
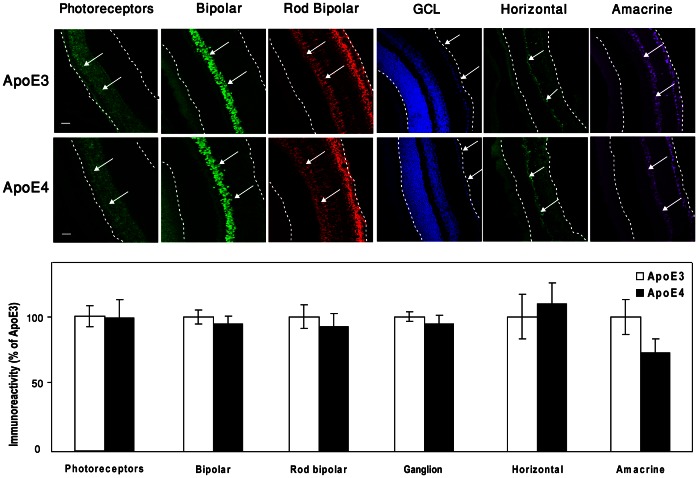
Immunostaining of distinct neuronal populations of the retina of apoE3 and apoE4 mice. Retinal sections were stained with the following neuron-specific markers: Recoverin (photoreceptors), CHX10 (pan bipolar cells), PKCα (rod bipolar), DAPI (ganglion cells), Calbindin (Horizontal cells), and PAX6 (amacrine cells) as described in Materials and Methods. Representative sections are presented in the upper panel. Retinal borders are marked with white dashed lines, and the specific cells are marked with arrows. Scale bar = 50 µm. The results (mean ± SEM, n = 10) are shown in the lower panel and are presented relative to the apoE3 mice whose values were set as 100%.

Next, we examined the possibility that the levels of distinct subpopulations of retinal neurons are affected by apoE4. This was performed immunohistochemically utilizing cell-specific markers, as described in Materials and Methods. As shown in figure 2, the levels of the photoreceptor and ganglion cells and of the bipolar cells that link them were the same in the apoE4 and apoE3 mice. The levels of the modulatory horizontal and amacrine cells were also not significantly affected by apoE4.

**Figure 3:**
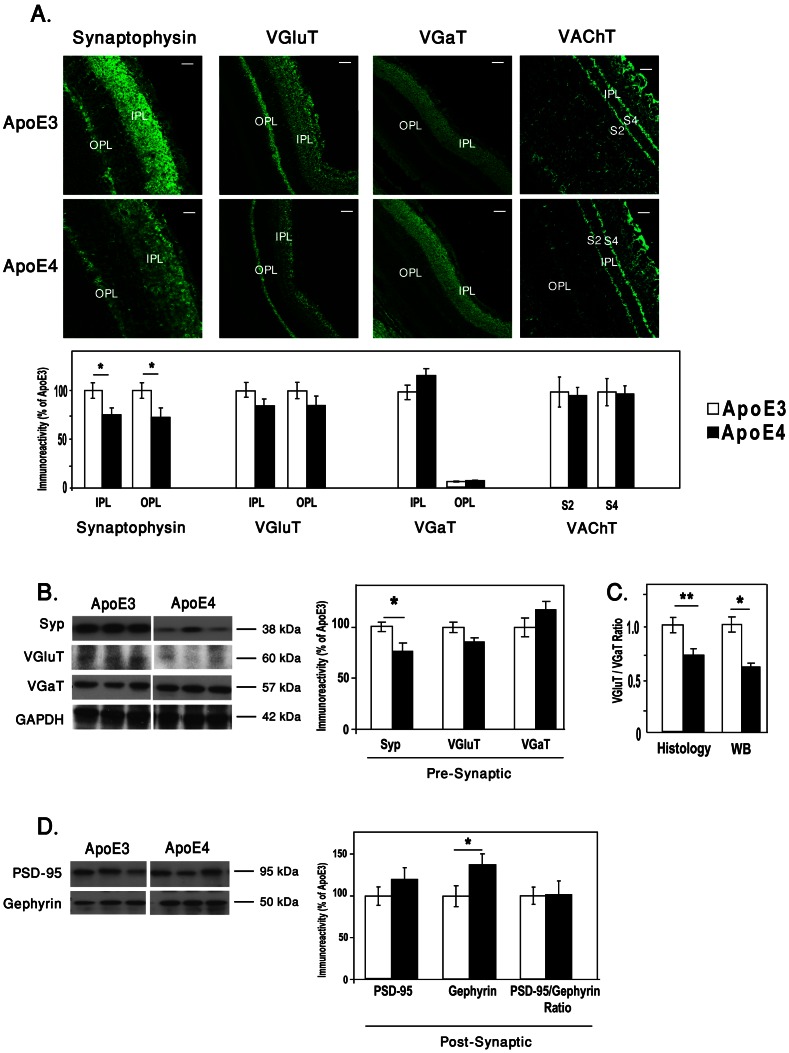
The effects of apoE4 on retinal nerve terminals. (A) Immunohistohemistry of retinal sections that were stained by the pan presynaptic marker synaptophysin and for the glutamatergic, GABAergic and cholinergic presynaptic vesicular transporters VGluT1, VGaT and VAChT, respectively, as described in Materials and Methods. Representative sections are depicted on the upper panel. Scale bar = 80 µm for Synaptophysin and 50 µm for VGluT1, VGaT and VAChT. Quantification of the VGluT1, VGaT and VAChT results (mean ± SEM, n = 10) is shown in the lower panel, and is represented relative to the apoE3 mice whose values were set as 100%. (B) Immunoblots of synaptophysin (Syp), VGluT1, and VGaT of retinal homogenates of apoE3 and apoE4. Representative blots are depicted on the left panels, and quantification of these results (mean ± SEM, n = 10) relative to the apoE3 mice is depicted on the right panel. (C) The ratio of VGluT1/VGaT of each mouse in both immunohistochemistry and western blots. (*P<0.03, **P<0.001). (D) Immunoblots of the post-synaptic markers PSD-95 and Gephyrin, of retinal homogenates of apoE3 and apoE4. Representative blots are depicted on the left panels, and quantification of these results and the ratio of PSD95/Gephyrin of each mouse (mean ± SEM, n = 10) relative to the apoE3 mice is depicted on the right panel.

We next considered the possibility that retinal synapses, unlike their corresponding perikarya, are affected by apoE4. Immunohistochemical measurements of the synaptic density, utilizing the synaptic protein synaptophysin as a marker revealed, as previously described, pronounced staining in the IPL and OPL [35]. Quantification of this staining in the IPL and OPL revealed that the levels of synaptophysin staining were significantly lower in the apoE4 than in the apoE3 mice (Figure 3A, left panels). Similar results were obtained by immunoblot experiments, which revealed that the levels of the 38 kDa synaptophysin immunoblot band were lower in the apoE4 than in the corresponding apoE3 mice (Figure 3B). The neuronal specificity of this synaptic effect was next assessed both pre-synapticly and post-synapticly. The pre-synaptic measurements were performed utilizing the pre-synaptic vesicular transporters VGluT1, VGaT and VAChT. The former is a marker of glutamatergic nerve terminals whereas VGaT resides in both GABAaergic and Glycinergic nerve terminals and VAChT resides in chiolinergic nerve terminals. This revealed, in accordance with the literature [36]–[38], that IPL was stained by VGluT1, VGaT and VAChT (strata s2 and s4) and that the OPL was stained by VGluT1, very weakly by VGaT and not by VAChT.

(Figure 3A upper panel). Quantification of these results revealed reduced levels of VGluT1 staining in the IPL and OPL, and elevated levels of VGaT staining in the IPL of the apoE4 retina. No difference between apoE3 and apoE4 was observed in either the levels or spatial distribution of VAChT (Fig. 3A lower panel). Similar results were obtained when staining of inner and outer halves of the IPL were analyzed separately. Complementary immunoblot experiments revealed, in accordance with the histological results, that apoE4 is associated with lower levels of VGluT1 and higher levels of VGaT (Figure 3B). Presentation of both the immunohistochemical staining and the immunoblot results in terms of the ratio of VGluT1/VGaT of each retina, revealed that this ratio decreased significantly in the apoE4 mice relative to the apoE3 mice in both assay systems (Figure 3C). Further analysis revealed that the ratio of VGluT1/synaptophysin of each retina was (1.0±0.13) and (1.04±0.19) for apoE3 and apoE4, respectively, and the ratio of VGaT/synaptophysin was (1.0±0.09) and (1.3±0.23) for apoE3 and apoE4, respectively. These results suggests that the decreased ratio of VGluT1/VGaT in the apoE4 (Figure 3C) is driven mainly by a decrease in VGluT1, similar to that of synaptophysin.

Post-synaptic measurements were assessed utilizing the post-synaptic scaffold protein PSD-95 as a marker of the excitatory synapses and the post-synaptic scaffold protein Gephyrin as a marker of the inhibitory synapses. This revealed increases in both PSD-95 and Gephyrin levels which was, however, significant only for Gephyrin (Fig. 3D). Importantly, the ratio of PSD-95/Gephyrin, unlike the ratio of VGluT/VGaT, is not affected by apoE4 (Fig. 3D), suggesting that the pre-synaptic effects of apoE4 are more pronounced than the corresponding post-synaptic effects.

**Figure 4:**
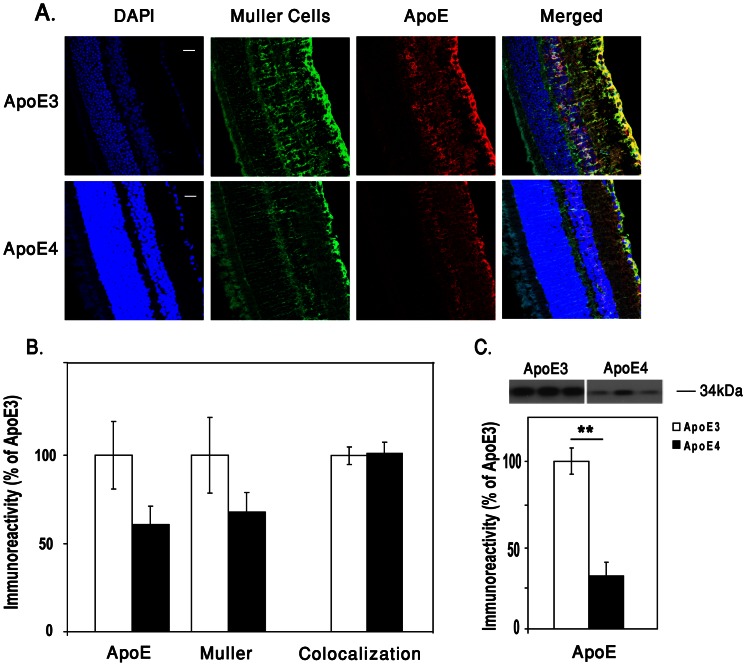
The effects of apoE4 on retinal apoE. (A) Immunohistochmistry. Representative images of retinal sections of both apoE3 and apoE4 stained for cell nuclei (DAPI - blue), GS (green), apoE (red), and the merged image. Scale bar = 50 µm. (B) Quantification of the GS, apoE and their co-localization. Results presented (mean ± SEM, n = 10) are relative to the apoE3 mice whose values were set as 100%. (C) ApoE immunoblot assays. Representative immunoblots are shown in the upper panel, whereas quantification of the results relative to the apoE3 mice (mean± SEM, n = 11) is presented in the lower panel. The immunoblot assays were performed as described in Materials and Methods. *P<0.0001.

The effect of apoE4 on the localization and expression levels of apoE in the retina was next examined. ApoE localization was determined by immunohistochemical double staining for apoE and the Muller cells marker glutamine synthetase (GS) (see Materials and Methods). This revealed, in accordance with previous publications [39]–[41], that apoE was localized in Muller cells. This was observed in both apoE3 and apoE4 retinas (Figure 4A). The levels of apoE, as assessed by both immunohistochemistry and western blot, were lower in the apoE4 than in the apoE3 mice (Figure 4B,C; P<0.001), as previously seen in the brain [42]. The quantification of the Muller cells marker, GS, revealed that the levels of GS were lower in apoE4 than in apoE3 retinas (Figure 4B).

**Figure 5:**
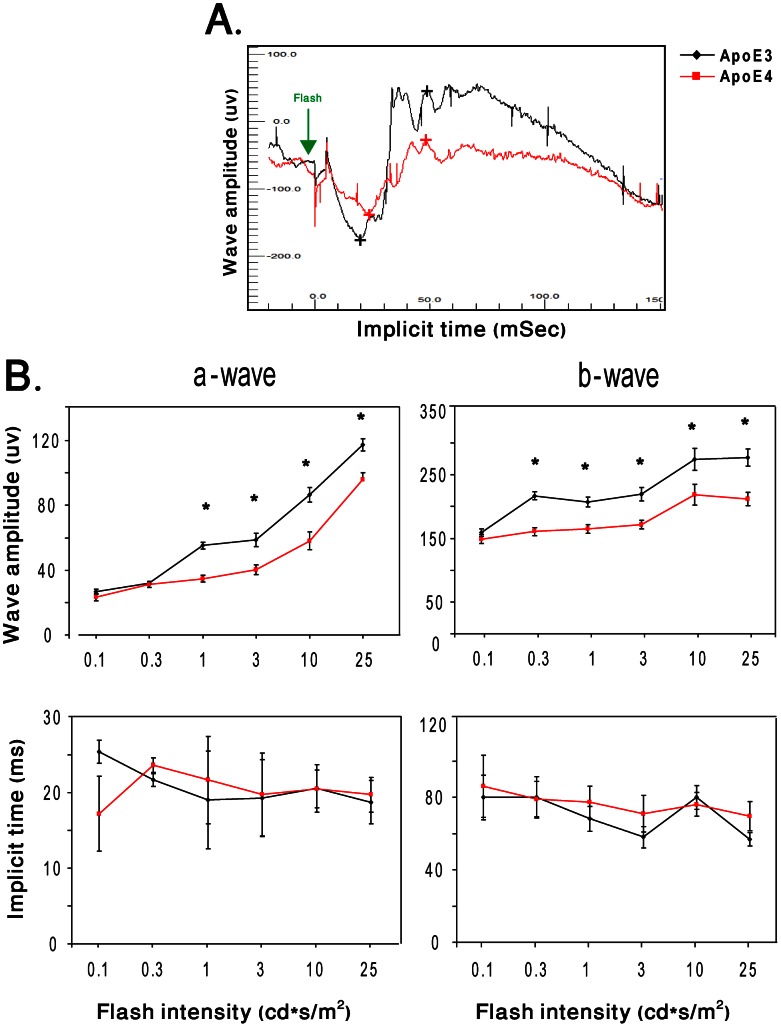
The effects of the apoE genotype on retinal function. Dark-adapted ERG responses of apoE3 and apoE4 mice were recorded in 13 steps (0.00003–25 cd*s/m^2^). (A) Representative ERG plots for apoE3 and apoE4 in response to a light flash. The peaks of a- and b-waves of apoE3 and apoE4 retinas are marked with black and red (+) signs, respectively. (B) Intensity response curves of the 6 highest light stimulation intensities (0.1, 0.3, 1, 3, 10, and 25 cd*s/m^2^) are presented. Results shown represent the mean ± SEM of the amplitudes (µV) and implicit times (ms) of a- and b-waves as a function of stimulus intensity. (n = 10; *P<0.05).

The effect of apoE4 on retinal function was measured using ERG. In the dark adapted mice, no significant differences were detected between apoE4 and apoE3 at the first seven luminance intensity levels (0.00003–0.03 cd*s/m^2^). In contrast, the a- and b-wave amplitudes of apoE4 mice were significantly lower than those of apoE3 mice at the higher light intensities (Figure 5B; upper panel; P<0.05). The ITs of both a- and b-waves were, however, not affected by mouse genotypes (Figure 5B; lower panel). Unlike in dark-adapted mice, the response of light adapted mice was not significantly affected by the apoE genotype.

## Discussion

This study investigated the extent to which the mouse retina is affected by apoE4 at a young age. Immunohistochemical studies revealed that the overall structure of the retina and the corresponding density of the perikarya of the different classes of retinal neurons were not affected by apoE4. In contrast, the synaptic density of the retinal IPL and OPL layers, as assessed immunohistochemically and by immunoblot experiments, was significantly lower in the apoE4 than in the apoE3 mice. This was associated with reduction of the ratio of the pre-synaptic parameters VGluT1/VGaT, which was mostly due to the reduced VGluT1 levels. The levels of the post-synaptic markers PSD-95 and gephyrin were increased in the apoE4 retinas, but their ratio was, however, not affected. ERG experiments revealed that mixed rod-cone responses were significantly lower in apoE4 relative to the apoE3 mice. Taken together, these findings show that apoE4 induces both histological and functional retinal impairments and suggest that the reduced ERG response may be related to the observed synaptic pathology.

The finding that the levels of the retinal glutamatergic transporter VGluT1 are specifically decreased by apoE4, is in accordance with our recent observation that apoE4 also decreases the levels of VGluT1 in the hippocampus of the apoE4 mice (in preparation). This observation is in agreement with findings in AD patients in which VGluT1 as well as other glutamatergic molecules and glutamatergic transmition are impaired [43]. It remains to be determined whether other glutamatergic pre-synaptic parameters in the retina of apoE4 mice, are also affected.

The mechanism underlying the glutamatergic effect of apoE4 is not fully understood. The finding that the levels of the apoE protein in the retina of apoE4 are lower than that of apoE3 (Fig. 4) was also observed in the hippocampus and other brain areas [42], [44] and may be due to increased degradation of apoE4 [44]. Since the levels of retinal apoE4 are lower than that of apoE3, it is possible that the retinal and brain synaptic susceptibility of the apoE4 mice is mediated via a loss of function mechanism. However, since some brain pathological effects of apoE4 seem to be mediated via a gain of toxic function (e.g., the synergistic cross talk between apoE4 and Aβ in brain neurons) [45], it is also possible that gain of toxicity mechanisms play a role in mediating the retinal effects of apoE4.

Recent findings suggest that the apoE receptor apoER2 plays an important role in the maintenance of retinal synaptic connections and promotes presynaptic differentiation and dendritic spine formation [46], [47]. Furthermore, it has been shown that apoE4 can reduce glutamate receptor function and synaptic plasticity via an apoER2-mediated mechanism [48]. It is thus possible that the presently observed specific vulnerability of the glutamatergic nerve terminals to apoE4 is mediated via apoER2. However, since the apoE receptor LRP, which resides on retinal Muller (glial) cells and vasculature [49], also responds isoform specifically to apoE4 [50], it may also play a role in mediating the effects of apoE4. In the brain, apoE4 interacts synergistically with Aβ [34], [43], [51], [52] and it remains to be determined whether similar mechanisms also mediate the effects of apoE4 in the retina.

The functional implication of the glutamatergic pathology in the retina was measured by ERG. This revealed that apoE4, in addition to its morphological and molecular effects also reduces the responsiveness of the dark-adapted retina to higher intensity light stimuli. These findings suggest that both rod and cone pathways are affected by apoE genotype. The lack of significant functional attenuation, of the response to dim flashes and under photopic conditions by apoE4, could result from minimal changes in each of the photoreceptor pathways, which lead to detectable changes only when the mixed rod-cone responses are recorded together. The fact that both a- and b-waves were affected by apoE4 implies an insult at the level of the photoreceptors (rods and cones) or at the level of both the photoreceptors and bipolar cells. A possible explanation for this finding may be a decrease in excitatory synapses along the rod and cone pathways. Photopic responses were generally similar in the apoE3 and apoE4 mice. The performance and behavior of apoE3 and apoE4 in open field and object recognition tasks, which are performed under dim light, were similar (not shown), suggesting that operationally speaking, the vision of the apoE4 mice was not impaired.

The mechanism underlying the effect of apoE4 on the levels of Muller cell marker GS is not known. Both GS and apoE are produced by the Muller cells, it is thus possible that the decrease in GS is due either to intracellular interactions between GS and apoE4 or to extra-cellular driven effects of the secreted apoE4. Additional studies, are required to assess the extent to which apoE4 also affects other glia components.

Paradoxically, and unlike in AD, apoE4 is protective in AMD, the leading cause of visual impairment and blindness among the elderly in western countries [29], [30], [53], [54]. The present findings and previous studies with mice maintained on a high cholesterol diet [55], both revealed pathological effects of apoE4. These include the presently reported apoE4 synaptic pathology in young naïve mice retinas and the formation of drusen like deposits in the cholesterol fed aged apoE4 mice. The finding that no protective effect of apoE4 was observed so far in mice can be due either to the scope of the paradigms used so far (e.g. lack of focus on neovascularization) or to inherent differences between the mouse and human retina (e.g. lack of macula in the mouse).

In conclusion, the present findings show that retinal synapses, like brain synapses, are affected by apoE4 in young mice. The finding that the effects of apoE4 on the retina and the brain are similar, along with the unique advantages of using the eye for imaging studies, suggests that the retina is an excellent system for non-invasive monitoring of the effects of apoE4 on the CNS in humans and in animal models and for the development of potential anti-apoE4 treatments.
